# Ureterovesical Junction and Waldeyer's Sheath: A Narrative Review Applied to Vesicoureteral Reflux

**DOI:** 10.1590/S1677-5538.IBJU.2026.9903

**Published:** 2026-03-10

**Authors:** Luciano A. Favorito, Ricardo C. de Mattos

**Affiliations:** 1 Universidade do Estado do Rio de Janeiro - Uerj Unidade de Pesquisa Urogenital Rio de Janeiro RJ Brasil Unidade de Pesquisa Urogenital - Universidade do Estado do Rio de Janeiro - Uerj, Rio de Janeiro, RJ, Brasil

**Keywords:** Vesico-Ureteral Reflux, Embryology, Review [Publication Type]

## Abstract

The ureterovesical junction (UVJ) is a specialized anatomical and functional unit responsible for maintaining unidirectional urinary flow from the ureter into the bladder. Dysfunction at this junction may lead to vesicoureteral reflux (VUR), obstruction, recurrent infections, and potential renal damage. The aim of this review is to present a narrative overview of the historical evolution in the understanding of the UVJ and to summarize key surgical principles, techniques, and contemporary controversies in its management. A narrative analysis of historical anatomical literature, landmark surgical innovations, and contemporary management strategies for UVJ pathology was performed. Emphasis was placed on seminal contributors and the evolution of surgical techniques. Early anatomical descriptions established the importance of the intramural ureter and trigonal anatomy. With the advent of cystography, VUR became recognized as a major pediatric urologic condition. Despite technological advances, the fundamental surgical principles governing UVJ reconstruction remain unchanged: adequate submucosal tunnel length, preservation of vascularity, and tension-free reimplantation. Ongoing advances in minimally invasive surgery and biomaterials continue to refine management strategies.

## INTRODUCTION

Primary vesicoureteral reflux (VUR) is a common pediatric condition that can lead to recurrent urinary tract infections, renal scarring, and reflux nephropathy (1). The ureterovesical junction (UVJ) is the point where each ureter enters the urinary bladder at its posterolateral aspect and traverses the bladder wall obliquely before opening at the ureteral orifice within the trigone. The ureterovesical junction plays a fundamental role in maintaining urinary continence and preventing VUR. The competence of the antireflux mechanism depends largely on the length of the intravesical ureter, its relationship to ureteral diameter, and the structural integrity of the bladder trigone (2). Developmental or structural abnormalities of this region have been associated with VUR and other functional disorders of the urinary tract (3).

The anatomical configuration of the distal ureter is fundamental to the competence of the antireflux mechanism. During fetal life, the relationship between intravesical ureteric length and ureteral diameter evolves, and shorter intravesical segments have been associated with VUR (4). Histological studies in patients with VUR have demonstrated smooth muscle disorganization, increased extracellular matrix deposition, and vascular alterations within the ureteral wall (5, 6).

This narrative review traces the historical milestones in the anatomical understanding of the UVJ and evaluates the importance of its embryological development and structural organization, including Waldeyer's sheath, with a focus on its role in vesicoureteral reflux and surgical innovation.

## EMBRIOLOGY APPLIED TO URETEROVESICAL JUNCTION

**Figure 1:**
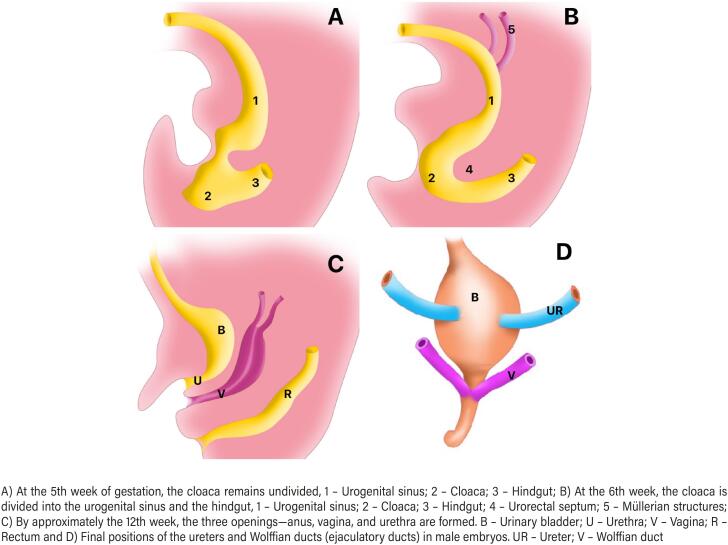
Embryological development of the cloaca and urogenital sinus.

The primitive cloaca is divided by the urorectal septum from the fourth to the seventh week after conception (7–9). The cloaca is divided into two parts: the anorectal canal (posterior) and the urogenital sinus (anterior) (Figure-1). The cloacal membrane is also divided into two parts: anteriorly, the urogenital membrane, and posteriorly, the anal membrane. The urogenital sinus that arises from the primitive cloaca is divided into three parts: the vesical, pelvic, and phallic portions.

A) At the 5th week of gestation, the cloaca remains undivided, 1 – Urogenital sinus; 2 – Cloaca; 3 – Hindgut; B) At the 6th week, the cloaca is divided into the urogenital sinus and the hindgut, 1 – Urogenital sinus; 2 – Cloaca; 3 – Hindgut; 4 – Urorectal septum; 5 – Müllerian structures; C) By approximately the 12th week, the three openings—anus, vagina, and urethra are formed. B – Urinary bladder; U – Urethra; V – Vagina; R – Rectum and D) Final positions of the ureters and Wolffian ducts (ejaculatory ducts) in male embryos. UR – Ureter; V – Wolffian duct

The vesical part is the most superior and largest portion of the urogenital sinus. Initially, it is continuous with the allantois, whose lumen later closes, giving rise to the urachus (7–9). The second part of the urogenital sinus, located below the vesical portion, is the pelvic part, which gives rise to the prostate and the membranous part of the urethra (Figure 1). The distal part of the urogenital sinus is the phallic portion, which is externally closed by the urogenital membrane (Figure-1).

Around the fifth week of development, the distal part of the mesonephric duct in relation to the ureteric buds dilates and is absorbed into the region of the urogenital sinus (8). The mesonephric ducts merge at the midline and form a triangular region, the future bladder trigone (10).

The bladder is embryologically divided into two portions: the body and the trigone. The bladder body derives from the endoderm of the vesical region of the urogenital sinus. The epithelium of this region is derived from the urogenital sinus endoderm, whereas the lamina propria, muscular layers, and adventitia derive from the adjacent splanchnic mesenchyme (7–9).

The fetal bladder can be identified by the tenth week postconception, due to the onset of urine production (11). The bladder is formed from mesenchymal and endodermal cells (11). Most of the urinary bladder originates from the vesical part of the urogenital sinus, while the trigone results from the absorption of the caudal region of the mesonephric duct during bladder development (11, 12). During the second gestational trimester, the bladder undergoes a series of developmental changes, ultimately acquiring a urothelial lining and a well-developed muscular coat (11–13).

The bladder trigone originates from the incorporation of the mesonephric ducts at the base of the developing bladder (10). Initially, these ducts contribute to forming the bladder trigone mucosa. However, this epithelium is later replaced by the endodermal epithelium of the urogenital sinus (7–9).

From the beginning of development until the 35th day after conception, the ureter is patent along its entire length (8). However, between the 37th and 40th days after conception, the ureteral lumen closes, and only its middle portion remains patent (8). After the 40th day, the ureteral lumen rapidly reopens both cranially and caudally, and the ureter becomes fully patent again.

Anomalies in ureteral development occur in approximately 10% of urological patients (14). The most common anomalies include partial and complete duplication, ectopic orifices, ureterocele, ureterovesical junction incompetence causing ureteral reflux, and intrinsic ureteral obstruction. Congenital ureteral obstructions occur most frequently at the pyeloureteral and ureterovesical junctions. According to Alcaraz (15), congenital ureteral obstructions may be divided into two major groups: intrinsic ureteral obstructions and obstructions of the ureterovesical junction. The latter occur due to the persistence of the ureterovesical membrane (Chwalla membrane), which temporarily obstructs this region between the 37th and 43rd days after conception (16).

Some bladder pathologies exhibit different patterns between the sexes, particularly primary vesicoureteral reflux, which is generally more severe in male fetuses and is associated with thickening of the bladder wall (17). The second trimester is particularly important in bladder embryonic development (11). The development of the prostate during the second gestational trimester, associated with hormone production by the fetal testes, may contribute to transient urethral obstruction in male fetuses (18, 19).

Muscle development in the region between the two ureteral orifices in the fetal bladder remains a subject of discussion, although it represents a fundamental morphological and functional component of the antireflux mechanism. This mechanism, which prevents urinary reflux, matures during the last trimester of gestation. The formation and proper configuration of this muscular network between the ureteral orifices and the bladder are essential for effective antireflux function (20).

## THE URETEROVESICAL JUNCTION

**Figure 2:**
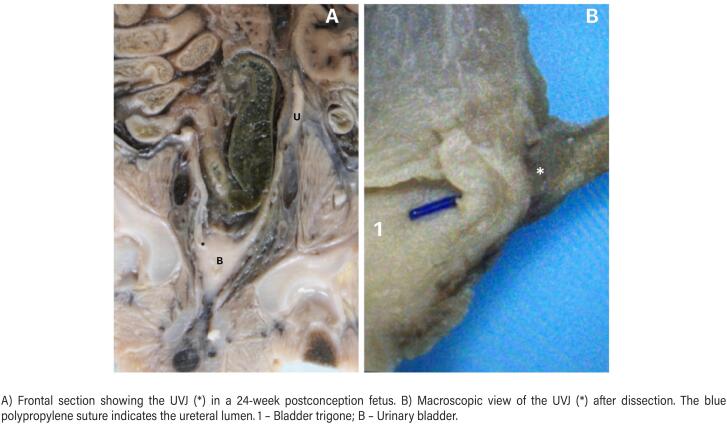
Ureterovesical junction (UVJ) in a human fetus.

**Figure 3:**
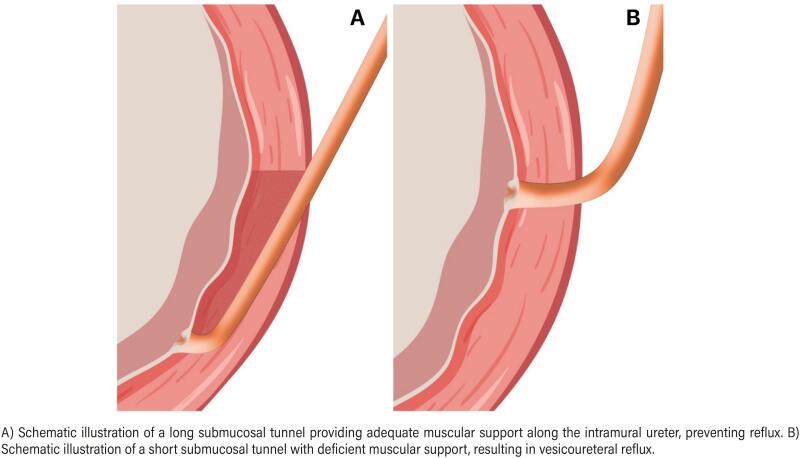
Schematic representation of the ureterovesical junction and the mechanism of vesicoureteral reflux. Defects of the ureterovesical junction may result in vesicoureteral reflux.

The ureterovesical junction represents the distal integration of the upper urinary tract into the bladder and functions as a dynamic antireflux mechanism (Figure-2). The portion of the ureter that passes through the bladder muscle layer is called the intramural segment; this portion of the ureter extends toward the bladder trigone. The bladder muscular layer that surrounds the intramural portion of the ureter, together with Waldeyer's sheath (a fibrous sheath that envelops the distal portion of the ureter), compose the mechanism responsible for preventing vesicoureteral reflux (21). Several conditions may cause vesicoureteral reflux, and one of the most common is anomalous implantation of the intramural ureter within the bladder. In patients with reflux, the morphology of the ureteral ostium at the vesical trigone is altered (Figure-3) (21).

A) Frontal section showing the UVJ (*) in a 24-week postconception fetus. B) Macroscopic view of the UVJ (*) after dissection. The blue polypropylene suture indicates the ureteral lumen. 1 – Bladder trigone; B – Urinary bladder.

A) Schematic illustration of a long submucosal tunnel providing adequate muscular support along the intramural ureter, preventing reflux. B) Schematic illustration of a short submucosal tunnel with deficient muscular support, resulting in vesicoureteral reflux.

Functionally, the UVJ acts as a dynamic antireflux mechanism, protecting the kidneys from elevated bladder pressures. Pathology at this junction manifests primarily as VUR or obstruction, both of which may lead to renal injury if left untreated. The understanding of UVJ anatomy and function has progressed from early cadaveric descriptions to advanced imaging techniques and minimally invasive reconstructive surgery.

The anatomical configuration of the UVJ produces a physiological valve mechanism: during bladder filling, the ureter remains patent, and during micturition, detrusor contraction compresses the intramural ureter, thereby preventing vesicoureteral reflux. Therefore, histologically, it functions as a dynamic muscular, sphincter-like support rather than a true anatomical sphincter.

**Figure 4:**
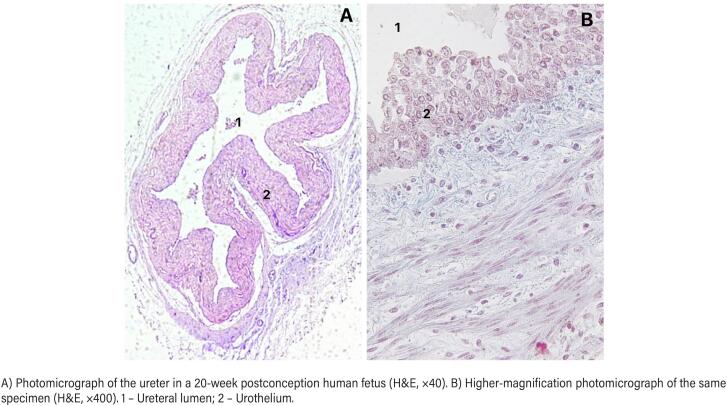
Histological features of the human ureter.

**Figure 5:**
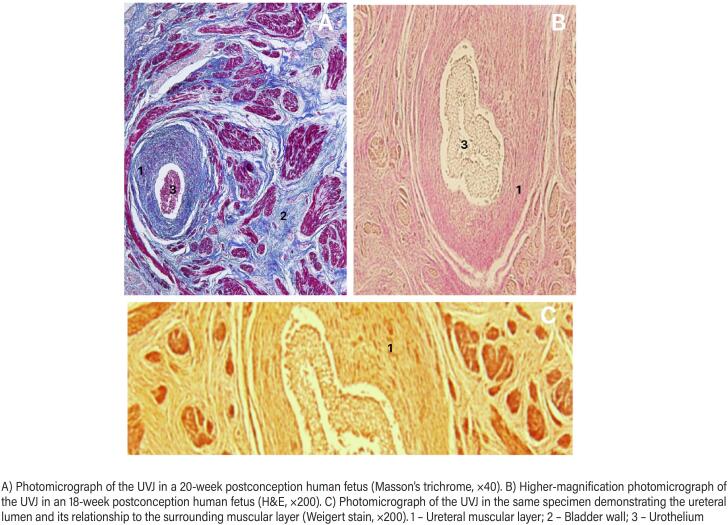
Histological features of the ureterovesical junction (UVJ).

Previous studies have described the histology of the ureter during the fetal period, demonstrating that the arrangement of the urothelium and the musculature of the ureteral wall is clearly evident during development (22) (Figure-4). In our laboratory, we performed a histological study of the UVJ and demonstrated the relationship between the ureter and the detrusor muscle (Figure-5).

A) Photomicrograph of the ureter in a 20-week postconception human fetus (H&E, ×40). B) Higher-magnification photomicrograph of the same specimen (H&E, ×400). 1 – Ureteral lumen; 2 – Urothelium.

A) Photomicrograph of the UVJ in a 20-week postconception human fetus (Masson's trichrome, ×40). B) Higher-magnification photomicrograph of the UVJ in an 18-week postconception human fetus (H&E, ×200). C) Photomicrograph of the UVJ in the same specimen demonstrating the ureteral lumen and its relationship to the surrounding muscular layer (Weigert stain, ×200). 1 – Ureteral muscular layer; 2 – Bladder wall; 3 – Urothelium

The caliber of the ureter along its course is not uniform, ranging from 2 to 10 millimeters in diameter. Along its course, there are three regions of physiological narrowing: the pyeloureteral junction, the crossing over the iliac vessels, and the intramural portion. These constriction points are common sites where calculi and blood clots may become lodged, and where the introduction of ureteroscopic instruments should be performed with caution to avoid perforation (23).

The blood supply to the ureter at the ureterovesical junction derives primarily from branches of pelvic vessels. In males, these include the superior vesical artery (a branch of the internal iliac artery), the inferior vesical artery, and occasionally the middle rectal artery. In females, the vascular supply includes the superior vesical artery, the uterine artery, and the vaginal artery. These arteries form a longitudinal anastomotic network along the ureter.

Venous drainage of the UVJ occurs through a ureteric venous plexus that drains into the vesical venous plexus and, in females, into the uterine or vaginal plexus, ultimately reaching the internal iliac vein. Lymphatic drainage of the UVJ occurs primarily to the internal iliac lymph nodes, with additional drainage to the external iliac nodes (23).

The ureterovesical junction receives autonomic innervation from the inferior hypogastric (pelvic) plexus. Sympathetic fibers originate from the T11–L2 spinal segments and travel via the lumbar splanchnic and hypogastric nerves. These fibers function to facilitate ureteric peristalsis and mediate pain (ureteric colic). Parasympathetic fibers originate from the S2–S4 spinal segments and travel via the pelvic splanchnic nerves, assisting peristalsis of the distal ureter.

## HEINRICH VON WALDEYER

**Figure 6:**
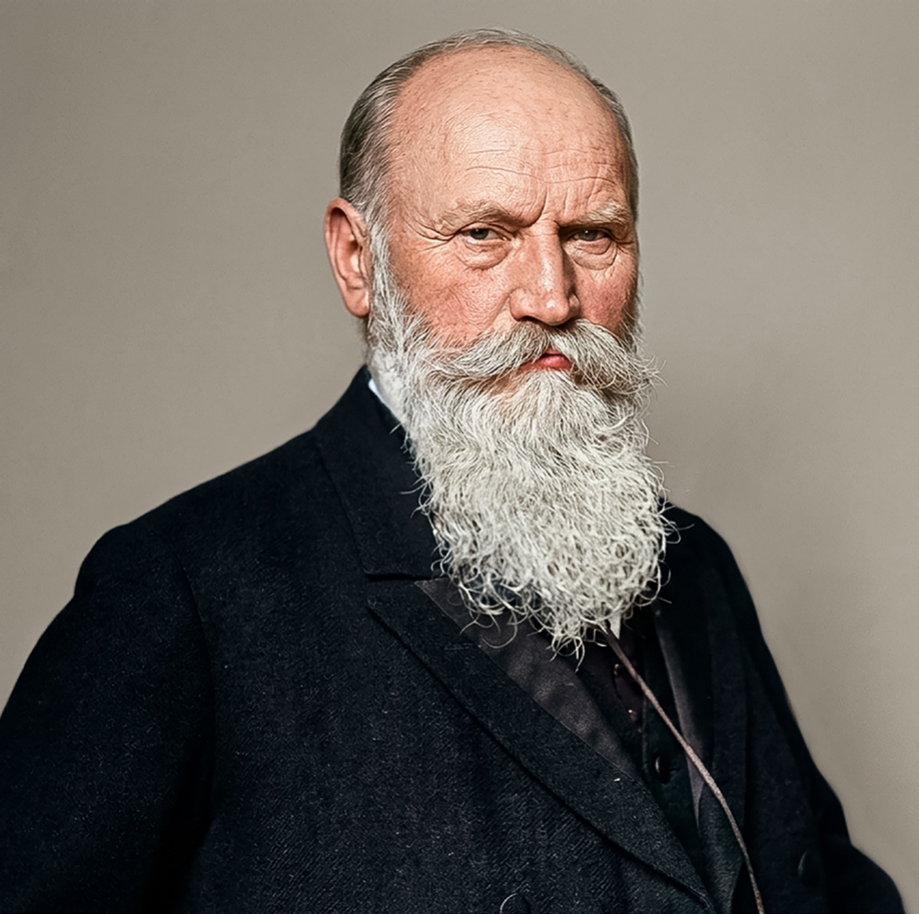
Original drawing of Heinrich von Waldeyer (1836–1921).

Heinrich Wilhelm Gottfried von Waldeyer-Hartz (1836–1921) was a German anatomist known for synthesizing neuron theory and for introducing the term "chromosome" (Figure-6). He is also remembered for anatomical structures of the human body that bear his name, including Waldeyer's tonsillar ring (the lymphoid tissue ring of the naso- and oropharynx) and Waldeyer's sheath. Waldeyer was one of the most prominent anatomists and scientists of his time and published 269 papers throughout his career. He also authored two important monographs, The Pelvis and Ovary and Egg. Numerous anatomical terms remain associated with his name (24).

In a brief report, Waldeyer described the presence of an injectable space beneath the musculature of the UVJ that allows this layer to be easily separated from the ureter, and he referred to a sheath-like structure, suggesting a gradual origin for Bell's muscle and other muscular components (25). Tanagho and Pugh (26) described "Waldeyer's sheath" in detail, stating that the sheath forms an encircling layer around the distal 1.5 to 3 cm of the ureter and consists histologically of fibromuscular tissue.

## THE WALDEYER’S SHEATH

Waldeyer's sheath is a fibromuscular extension of the bladder detrusor muscle that surrounds the intramural ureter, anchoring the ureter within the bladder wall, contributing to the antireflux valve mechanism, and coordinating with detrusor contraction during voiding. Waldeyer's sheath develops as the detrusor muscle differentiates from the splanchnic mesoderm. As the trigone forms and the ureters are incorporated into the bladder wall, muscle fibers reorganize around the intramural segment, forming Waldeyer's sheath.

Waldeyer's sheath contains collagen and elastic fibers and is composed predominantly of detrusor-derived smooth muscle. It surrounds the distal muscularis and blends with the bladder detrusor, whereas the ureter at the UVJ contains urothelium, lamina propria, and muscularis (inner longitudinal, outer circular, and an additional outer longitudinal layer distally).

The intramural ureter within Waldeyer's sheath provides structural support to the antireflux mechanism and does not function as a true anatomical sphincter (26–28).

Waldeyer and colleagues in 1892 (24) were the first to describe the interureteral muscular junction, referred to as the "torus interureteralis," although the term "interureteral crest" later became more widely adopted. Some authors have described the trigone as a muscular formation resulting from the convergence of ureteral muscle fibers that descend in a fan-shaped pattern, with greater concentration at the lateral border (26). Others suggest that most ureteral smooth muscle fibers blend contralaterally, forming an interureteral ridge, of which only a portion contributes to the prominence of the lateral border (27). A layer of smooth muscle derived from Waldeyer's sheath, located beneath the so-called superficial trigone, has been described as constituting the "deep trigone" (26, 29).

The concept of Waldeyer's sheath and the structure of the deep trigone were revised following a reassessment of the anatomy of the ureterovesical junction (28, 30, 31). More recently, the existence of an independent vesical muscular sphincter has been postulated, which is thought to traverse the internal urethral orifice in an elliptical manner (32, 33).

**Figure 7:**
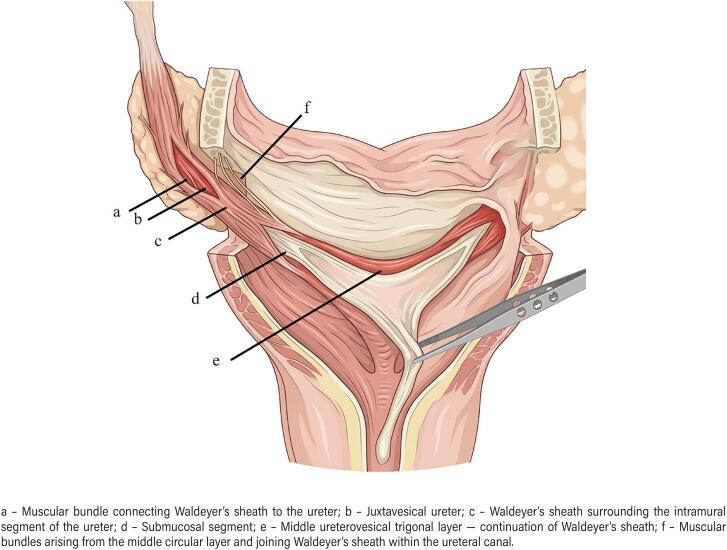
Anatomical relationships of Waldeyer's sheath at the ureterovesical junction.

Detailed anatomical studies conducted by Tanagho and Pugh (26) confirmed, through dissections and serial histological sections, that this sheath constitutes a continuous mantle of fibromuscular tissue surrounding the juxtavesical and intravesical segments of the ureter. The authors demonstrated that it does not originate from the bladder musculature, as previously proposed, but rather represents an extension of the outer layers of the ureter (Figure-7).

a – Muscular bundle connecting Waldeyer's sheath to the ureter; b – Juxtavesical ureter; c – Waldeyer's sheath surrounding the intramural segment of the ureter; d – Submucosal segment; e – Middle ureterovesical trigonal layer — continuation of Waldeyer's sheath; f – Muscular bundles arising from the middle circular layer and joining Waldeyer's sheath within the ureteral canal.

Although most anatomical studies recognize that Waldeyer's sheath continues into the trigone of the bladder, there is disagreement regarding the designation of the anatomical layer with which it integrates. In the study by Tanagho and Pugh (26), the sheath is described as forming the middle layer of the trigone, located between the superficial layer (formed by submucosal ureteral fibers) and the deep layer (composed of the detrusor muscle). More recent descriptions refer to this same structure as the deep layer, considering three distinct planes: submucosal (superficial), Waldeyer's sheath (deep), and detrusor (basal). This therefore represents a terminological variation rather than a structural or embryological difference. Rusu et al. (34) highlighted the compartmental character of the sheath in the regulation of ureterovesical continence and observed that similar structures may be present along the proximal ureter, suggesting that this represents a common anatomical feature of the human ureter (35).

Functionally, Waldeyer's sheath contributes to the lengthening and stabilization of the intravesical ureter and, consequently, to the competence of the antireflux mechanism. The interaction between its fibers and the trigonal muscle complex facilitates proper anatomical positioning of the ureter and effective occlusion of the ureteral orifice during bladder filling and voiding. Tanagho and Pugh (26) described that the coordinated contraction of the superficial trigonal layer acts as an oblique sphincter-like mechanism, promoting collapse of the intravesical ureter by approximating its superior and inferior walls. Waldeyer's sheath reinforces this mechanism by stabilizing the ureter within the vesical hiatus and contributing to dynamic sealing of the orifice. These findings support the hypothesis that morphological or embryological alterations of this sheath—such as those occurring in central nervous system malformations or urogenital developmental disorders—may compromise the architecture and function of the bladder trigone, thereby predisposing to congenital urinary dysfunction.

Proper development of the trigone and ureterovesical junction is essential for maintaining continence and preventing vesicoureteral reflux. The configuration of the ureteral tunnel, its intramural extension, and adequate muscular support together provide a functional valve mechanism that permits unidirectional urine flow toward the bladder, thereby protecting the kidneys from retrograde pressure and ascending infection (36) (Figure-7).

The morphogenesis of these structures is critical for establishing normal urinary tract function. Disturbances in this embryological process may lead to structural alterations of the bladder and urethra, resulting in functional impairment (37), as demonstrated in cases of bladder exstrophy, in which urethral length plays a determining role in achieving continence and, consequently, in planning reconstructive surgical interventions (38).

A summary of the differences between the ureterovesical junction and Waldeyer's sheath is presented in Table-1.

**Table 1:** The table shows the differences between the ureterovesical junction and Waldeyer's sheath.

FEATURE	URETEROVESICAL JUNCTION	WALDEYER’S SHEATH
Definition	Junction of ureter & bladder	Fibromuscular covering of intramural ureter
Embryologic origin	Ureteric bud + trigone incorporation	Differentiated detrusor muscle
Main function	Prevent reflux, allow urine entry	Structural support & valve mechanism
Clinical link	VUR, megaureter, ureterocele	Anti-reflux failure if defective

## CONCLUSIONS

The evolution in the understanding and management of the ureterovesical junction reflects the broader trajectory of urologic surgery—from anatomical observation to reconstructive refinement and minimally invasive precision. Although surgical techniques continue to evolve, the fundamental anatomical principles governing antireflux function remain constant.

## Data Availability

All data generated or analysed during this study are included in this published article
